# Efficient haplotype block recognition of very long and dense genetic sequences

**DOI:** 10.1186/1471-2105-15-10

**Published:** 2014-01-14

**Authors:** Daniel Taliun, Johann Gamper, Cristian Pattaro

**Affiliations:** 1 Center for Biomedicine, European Academy of Bolzano/Bozen (EURAC), Bozen-Bolzano, Italy; 2 Free University of Bozen-Bolzano, Bozen-Bolzano, Italy

## Abstract

**Background:**

The new sequencing technologies enable to scan very long and dense genetic sequences, obtaining datasets of genetic markers that are an order of magnitude larger than previously available. Such genetic sequences are characterized by common alleles interspersed with multiple rarer alleles. This situation has renewed the interest for the identification of haplotypes carrying the rare risk alleles. However, large scale explorations of the linkage-disequilibrium (LD) pattern to identify haplotype blocks are not easy to perform, because traditional algorithms have at least *Θ*(*n*^2^) time and memory complexity.

**Results:**

We derived three incremental optimizations of the widely used haplotype block recognition algorithm proposed by Gabriel *et al*. in 2002. Our most efficient solution, called MIG ^++^, has only *Θ*(*n*) memory complexity and, on a genome-wide scale, it omits >80*%* of the calculations, which makes it an order of magnitude faster than the original algorithm. Differently from the existing software, the MIG ^++^ analyzes the LD between SNPs at any distance, avoiding restrictions on the maximal block length. The haplotype block partition of the entire HapMap II CEPH dataset was obtained in 457 hours. By replacing the standard likelihood-based *D*^′^ variance estimator with an approximated estimator, the runtime was further improved. While producing a coarser partition, the approximate method allowed to obtain the full-genome haplotype block partition of the entire 1000 Genomes Project CEPH dataset in 44 hours, with no restrictions on allele frequency or long-range correlations. These experiments showed that LD-based haplotype blocks can span more than one million base-pairs in both HapMap II and 1000 Genomes datasets. An application to the North American Rheumatoid Arthritis Consortium (NARAC) dataset shows how the MIG ^++^ can support genome-wide haplotype association studies.

**Conclusions:**

The MIG ^++^ enables to perform LD-based haplotype block recognition on genetic sequences of any length and density. In the new generation sequencing era, this can help identify haplotypes that carry rare variants of interest. The low computational requirements open the possibility to include the haplotype block structure into genome-wide association scans, downstream analyses, and visual interfaces for online genome browsers.

## Background

Linkage disequilibrium (LD) is the non-random association of alleles at different loci and decays with increasing distance between loci [1]. High LD regions reflect the presence of chromosomal segments (haplotypes) that are transmitted from parents to offsprings more often than expected by chance. LD is traditionally assessed by the normalized *D*^′^ coefficient [2]. The *r*^2^ coefficient [3] is currently more commonly used than *D*^′^ to identify independent signals in genome-wide association studies (GWAS). In this regard, the *r*^2^ was also considered in haplotype block recognition algorithms [4,5]. Nevertheless, the *D*^′^ should remain the statistics of choice for LD modeling because of its more direct biological interpretation. It reflects the history of recombination, mutation, and selection events that cause some chromosomal segments to be less diverse than others and, therefore, influence the haplotype distribution. Moreover, it has been shown that *r*^2^ is not significantly more precise, accurate or efficient than *D*^′^[6]. The *D*^′^ and *r*^2^ coefficients capture similar information but their range of variation can be very different [6]. The *D*^′^ goes from -1 to +1 and is independent of the allelic frequencies of the two markers involved. When *D*^′^ = 0, the two markers are independent (perfect equilibrium), while |*D*^′^| = 1 indicates that no more than three of the four possible haplotypes are being observed in the sample (complete disequilibrium). In contrast, the range of *r* greatly depends on the allele frequencies and equals -1 or +1 only when the two markers have the same allele frequency. In such cases, |*r*| = 1 indicates that knowing the allele at one marker allows to determine the allele at the other marker (perfect disequilibrium). But when the two markers have very different allele frequencies, the interpretation of *r*^2^ becomes difficult. This is especially relevant with the data generated by the new sequencing technologies, that allow genotyping markers over a very wide spectrum of allele frequencies. In such situations, the *r*^2^ may fail to identify the correct relationship between nearby variants. In GWAS, this may lead to a wrong definition of the identified loci.

Although, in the past, haplotype blocks have been mainly used to identify tag SNPs [7], a variety of other applications is possible with currently available data. Recently, analysis of exome-chip data has shown that within-gene LD-block distribution can be informative of the gene function and of the possible relationship between genes and specific groups of phenotypes [8]. Another application is the genome-wide haplotype association scan, which was successful in uncovering risk loci for coronary artery disease [9], Alzheimer’s disease [10] and breast cancer [11]. So far, genome-wide haplotype association scans have been mostly performed based on fixed- or variable-width sliding window methods, which systematically miss haplotypes that are longer than some specified maximal window widths. An efficient genome-wide haplotype block recognition could help overcome such limitations, thus enhancing the biological interpretation of the results. In the study of rare variants, where collapsing methods (mostly based on gene boundaries) are becoming increasingly popular [12], the availability of haplotype blocks at genome-wide level would allow to collapse variants based on block boundaries, capturing inter-genic variants, and avoiding the problem to define the gene boundaries. Additional applications include downstream analyses of GWAS, such as pathway-based approaches, where statistics for multiple SNPs are summarized into gene-specific P-values, which are then employed for gene ranking [13]. In pathway-based analyses, SNP-to-gene mapping is typically based on SNP proximity to the gene boundaries. With this method, when a region is gene-dense, it may be problematic to assign SNPs to a single, specific gene. An LD-based assignment would overcome this limitation and increase the power of downstream analyses [14]. In general, ignoring the LD structure in downstream analyses of GWAS can result in the misinterpretation of the findings [15]. Popular genome browsers, such as the Ensembl [16] or UCSC [17], are suitable for visualizing the LD distribution over regions of interest. However, they only allow pairwise LD calculation between markers at <500 kb distance from each other and do not provide any LD-block partition. With no predefined block partition, the visual assessment of such LD patterns might be influenced by investigator’s subjectivity. On the other hand, the 500 kb distance constraint may limit the investigation of larger strong-LD regions. With the availability of pre-calculated, threshold-free LD blocks, we would overcome both these limitations.

There is extensive literature on haplotype block inference [18-21], including methods based on probabilistic graphical models [22]. The latter allow an accurate identification of SNP clusters, even in situations when SNPs are not necessarily contiguous. However, due to its simplicity, the most commonly used LD-based algorithm remains the one proposed by Gabriel *et al.*[23], which is implemented in Haploview [24]. The Haploview algorithm is widely used in genetic association studies and it is included in popular software, such as PLINK [25]. However, with a *Θ*(*n*^2^) time and memory complexity, where *n* is the number of SNPs, the algorithm is applicable only to short genomic segments containing no more than a few thousand SNPs. Unless runtime and memory usage are artificially reduced by splitting large segments into smaller chunks, the algorithm cannot be applied to densely genotyped segments or genome-wide analyses.

In this paper, we describe how we improved efficiency and scalability of the Haploview algorithm (1) by adopting an incremental computation of the haplotype blocks based on iterative chromosome scans and (2) by estimating *D*^′^ confidence intervals (CIs) using the approximate variance estimator proposed by Zapata *et al.*[26]. The incremental computation strategy led to an algorithm, termed MIG ^++^, that has *Θ*(*n*) memory complexity and omits more than 80% of the pairwise LD computations, while obtaining exactly the same final haplotype block partition as Haploview. In contrast to Haploview, the new algorithm can consider pairwise LD between SNPs at any distance. With MIG ^++^, we performed the haplotype block recognition of the entire HapMap phase II dataset of CEPH haplotypes. By introducing the approximate variance estimator, the performance of the MIG ^++^ was further improved and allowed us to perform the block partition of the entire 1000 Genomes Project dataset of CEPH haplotypes. To show a practical application of the obtained genome-wide block partition, we finally compared SNP-based and haplotype block-based association tests in a GWAS context.

## Methods

### Haplotype block definition

The haplotype block recognition algorithm proposed by Gabriel *et al.*[23] is based on |*D*^′^| and its 90% CI, with CL and CU being the lower and upper bounds of the CI, respectively. SNP pairs are classified as follows: (1) in *strong LD* if *CL* ≥ 0.7 and *CU* ≥ 0.98; (2) *showing strong evidence of historical recombination* (*strong EHR*) if *CU*<0.9; (3) *non-informative*, otherwise. *Informative* pairs are those satisfying conditions (1) or (2). A haplotype block was then defined as follows:

#### 

**Definition 1. ***(Haplotype Block)*. *Let C* = 〈*g*_1_, …,*g*_*n*_〉 *be a chromosome of n SNPs*, *G* = 〈*g*_*i*_, …, *g*_*j*_〉 *a region of adjacent SNPs in C*, *l the number of strong LD SNP pairs in G*, *and r the number of strong EHR SNP pairs in G*. *Then*, *G is a haplotype block if*

*(a) the two outermost SNPs*, *g*_*i *_*and g*_*j*_, *are in strong LD*, *and*

*(b) there is at least a proportion d of informative pairs that are in strong LD*, *i.e.*: *l* / (*l* + *r*) ≥ *d*.

In their original work, Gabriel *et al.*[23] set *d* = 0.95 after investigating the fractions of *strong LD* SNP pairs in genomic regions of different length and in different populations.

The Haploview algorithm [24] performs a haplotype block partition in two steps: (1) all regions satisfying Definition 1 (a) are collected in a set of candidate haplotype blocks; (2) from this set of candidates, a subset of non-overlapping regions that satisfy Definition 1 (b) is selected. In the first step, the entire chromosome is scanned and, for every SNP pair, the |*D*^′^| CI is computed and stored in an *n* × *n* matrix. The matrix is then traversed to identify the pairs that satisfy Definition 1 (a). These pairs mark regions of different length that are candidates to become haplotype blocks. In the second step, the candidate regions are sorted by decreasing length and processed starting with the largest one. If a region satisfies Definition 1 (b), it is classified as a haplotype block, and all other overlapping candidate regions are discarded. Regions not satisfying Definition 1 (b) are skipped. This process continues with the next largest candidate region, until the candidate set is completely processed and the list of haplotype blocks is complete.

The overall complexity of the algorithm is mainly determined by the first step. More specifically, the *Θ*(*n*^2^) time and memory complexity is due to the computation and maintenance of the *n* × *n* CI matrix. For this reason, we concentrated our improvements on the first step.

### Incremental computation of haplotype blocks

The core ideas of our optimizations are to compute haplotype blocks incrementally and to omit, as soon as possible, regions that cannot be extended to larger blocks due to an insufficient proportion of *strong LD* SNP pairs. In this way, we avoid both unnecessary computations and the storage of an *n* × *n* CI matrix. The incremental haplotype block computation is based on the concepts of a *SNP-pair weight* and a *region weight* described below.

#### 

**Definition 2. ***(SNP-pair weight)*. *Let C and d be as defined in Definition 1. For a given pair of SNPs g*_*i *_*and g*_*j*_, *the SNP-pair weight*, *w*(*i*, *j*), *is defined as follows:*

$$w(i,j)=\left\{\begin{array}{l} 1-d\;\;\;\text{if}\;\;g_{i}\;\;\text{and}\;\;g_{j}\;\;\text{are in strong LD},\\ -d\;\;\;\text{if}\;\;g_{i}\;\;\text{and}\;\;g_{j}\;\;\text{show strong EHR},\\ 0\;\;\;\text{otherwise.} \end{array}\right)$$

#### 

**Definition 3. ***(Region weight)*. *Let G be as defined in Definition 1*. *The region weight of G*, $\bar{w}(i,j)$, *is defined as the sum of all SNP-pair weights in G*:

$$\begin{array}{l} \bar{w}(i,j)=\overset{j}{\underset{v=i+1}{\sum }}\overset{v}{\underset{u=i}{\sum }}w\;(u,v). \end{array}$$

The following theorem defines a haplotype block based on the region weight.

#### 

**Theorem 1**. *Let G be as defined in Definition 1. G is a haplotype block if w* (*i*, *j*) = 1 - *d and*$\bar{w}(i,j)\geq 0$.

#### *Proof*

From Definition 2, if SNPs *g*_*i*_ and *g*_*j*_ are in *strong LD*, then *w* (*i*, *j*) = 1 - *d*. Therefore, Definition 1 (a) is satisfied. *G* contains $S=\overset{j}{\underset{v=i+1}{\sum }}\overset{v}{\underset{u=i}{\sum }}1$ possible SNP pairs, of which *l* are in *strong LD*, *r* show *strong EHR*, and the remaining ones are non-informative. From Definitions 2 and 3, it follows that $\bar{w}(i,j)=\overset{j}{\underset{v=i+1}{\sum }}\overset{v}{\underset{u=i}{\sum }}w\;(u,v)=l(1-d)+r(-d)+(S-l-r)\cdot 0=l-d(l+r)$. If $\bar{w}(i,j)\geq 0$, then *l* - *d*(*l* + *r*) ≥ 0 ⇒ *l* / (*l* + *r*) ≥ *d*. Therefore, Definition 1 (b) is also satisfied.

Theorem 1 is the basis for the incremental haplotype block reconstruction, which is the core of our optimizations. In the following, we present three gradual improvements of the Haploview algorithm: a memory-efficient implementation based on the Gabriel *et al.*[23] definition (MIG); MIG with additional search space pruning (MIG ^+^); and MIG ^+^ with iterative chromosomal processing (MIG ^++^). Theorem 1 ensures that all three algorithms produce block partitions that are identical to the original Haploview results.

#### The MIG algorithm

**Figure 1:**
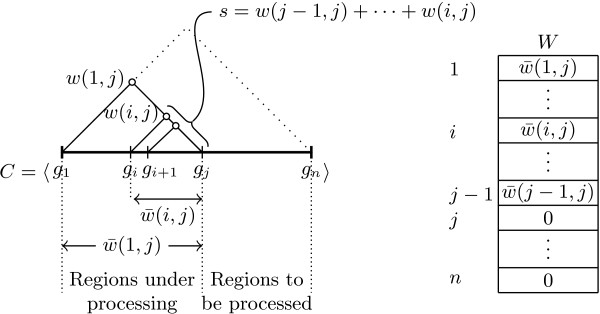
**Processing a chromosome with the MIG algorithm.** A chromosome is processed from left to right, and *g*_*j*_ is the current SNP. Values in *W* [*i*] correspond to the region weights $\bar{w}(i,j)$ of 〈*g*_*i*_, …, *g*_*j*_〉. *s* accumulates SNP-pair weights *w*(*j* - 1, *j*), …, *w*(*i*, *j*).

For a given chromosomal segment *C* containing *n* SNPs, the maintenance of an *n* × *n* matrix containing all the |*D*^′^| CIs can be avoided by storing *n* region weights in a unidimensional vector *W*_*n* × 1_. In each element of *W*, *W*[*i*], we store the weight of a chromosomal region that starts at SNP *g*_*i*_. When the region is enlarged by including additional SNPs to the right of *g*_*i*_, the weight *W*[*i*] is updated accordingly. This procedure, illustrated in Figure 1, begins with setting all the weights to 0. At the initial stage, the vector *W* represents all one-SNP regions. Then, the region starting at SNP *g*_1_ is enlarged by including the next SNP, *g*_2_. Therefore, starting from *g*_2_, chromosome *C* is processed one SNP after the other, from left to right. For a SNP *g*_*j*_, with *j* ≥ 2, all SNP pair weights *w*(*i*, *j*), *i* = *j* - 1, …, 1, are computed and added up as *s* = *w*(*j* - 1, *j*) + ⋯ + *w* (*i*, *j*).

**Figure 2:**
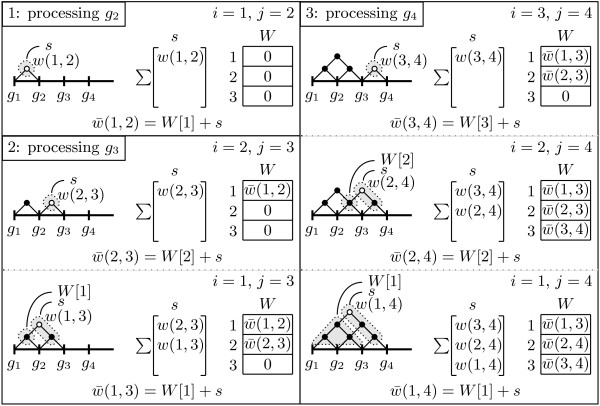
**The first three computational steps of the MIG algorithm.** The vector *W* and the variable *s* are initialized to 0. The processing starts at *g*_2_ with the analysis of the region 〈*g*_1_, *g*_2_〉. The SNP-pair weight *w*(1, 2) is computed and added to *s*. Then, the region weight $\bar{w}(1,2)$ is incrementally computed as *W*[1] + *s* and stored in *W*[1] (by replacing the old value). Next, SNP *g*_3_ is processed. After initializing *s* = 0, weight *w*(2,3) is computed and stored in *s*. $\bar{w}(2,3)$ is incrementally determined as *W*[2] + *s*, and stored in *W*[2]. Then, weight *w*(1,3) is computed, and $\bar{w}(1,3)=\bar{w}(1,2)+w(2,3)+w(1,3)=W[\;1]+s$. The next SNP to the right, *g*_4_, is processed in a similar way.

*s* and *W*[*i*] are updated for every computed weight *w*(*i*, *j*). Before the update, *s* = *w*(*j* - 1, *j*) + ⋯ + *w*(*i* - 1, *j*) and *W*[*i*] contains the region weight $\bar{w}(i,j-1)$, which was already computed for the previous SNP *g*_*j*-1_. Then, *s* is incremented by *w*(*i*, *j*) and *W*[*i*] is incremented by the new value of *s*. *W*[*i*] now represents the region weight $\bar{w}(i,j)$, i.e., $\bar{w}(i,j)=\bar{w}(i,j-1)+w(j-1,j)+\cdots +w(i,j)$. Whenever *w*(*i*, *j*) = 1 - *d* and $\bar{w}(i,j)\geq 0$, Theorem 1 is satisfied and the region 〈*g*_*i*_, …, *g*_*j*_〉 is added to the set of candidate haplotype blocks. This procedure is repeated with the next SNP, *g*_*j*+1_. An example of the first three computational steps is given in Figure 2. The pseudocode is provided in Algorithm A.1 (Additional file 1).

MIG reduces the memory complexity from *Θ*(*n*^2^) to *Θ*(*n*). Moreover, instead of identifying candidate regions that satisfy only Definition 1 (a) (as in Haploview), MIG checks immediately both conditions (a) and (b). This yields a smaller set of candidate blocks, and therefore indirectly speeds up also the second step of the Haploview algorithm.

#### The MIG ^+^ algorithm

While MIG drastically reduces the memory requirements by avoiding the maintenance of the CI matrix, it still computes weights for all SNP pairs, totaling *n*(*n* - 1) / 2 computations as in Haploview. To omit unnecessary computations, we apply a search space pruning to the MIG algorithm to identify regions that cannot be further extended to form a haplotype block. The pseudocode is shown in Algorithm A.2 (Additional file 1).

**Figure 3:**
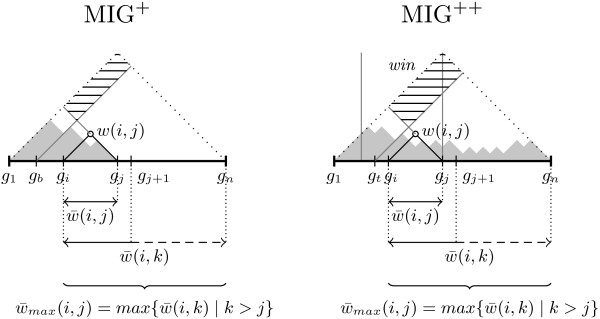
**Processing a chromosome with MIG**^**+ **^**and MIG**^**++**^**.**${\bar{w}}_{\text{max}}(i,j)$ is computed taking into account the SNP-pair weights computed at the previous stages (colored in gray). MIG ^+^: since ${\bar{w}}_{\text{max}}(i,j)<0$ for any region that starts before the SNP *g*_*b*_ and ends after the SNP *g*_*j*_, then the SNP-pair weights within the hatched area are omitted from computation. MIG ^++^: the SNP-pair weights within the hatched area are omitted from computation if ${\bar{w}}_{\text{max}}(i,j)<0$ for any region that starts before the SNP *g*_*t*_ and ends after the SNP *g*_*j*_, or *g*_*t*_ is out of the window *win* (in the latter case the omitted SNP-pair weights may be used in the next iteration).

Instead of computing weights for all pairs of SNPs, only weights *w*(*j* - 1, *j*), …, *w*(*b*, *j*) are computed, where $b=\text{min}(\{i|1\leq i<j\wedge {\bar{w}}_{\text{max}}(i,j)\geq 0\})$ and ${\bar{w}}_{\text{max}}(i,j)=\text{max}\{\bar{w}(i,k)|j<k\leq n\}$. The function ${\bar{w}}_{\text{max}}(i,j)$ is an upper bound for the weight of all regions 〈*g*_*i*_, …, *g*_*j*_, …, *g*_*k*_〉 that start at *g*_*i*_ and end after *g*_*j*_, i.e., those extending beyond the region 〈*g*_*i*_, …, *g*_*j*_〉. If ${\bar{w}}_{\text{max}}(i,j)<0$ for some *i*, none of the regions 〈*g*_*i*_, …, *g*_*k*_〉 can satisfy Definition 1 (b). The smallest *i*, that can be a potential starting point of a region with a positive weight, can therefore be set as breakpoint *b*. Regions starting left of *b* and stopping right of *j* receive negative weights and are discarded (Figure 3, left panel).

The upper bound, ${\bar{w}}_{\text{max}}(i,j)$, is estimated assuming that all unprocessed SNPs to the right of *g*_*j*_ are in *strong LD* with each other and with all SNPs in the region 〈*g*_*i*_, …, *g*_*j*_〉. Then, $\bar{w}(i,k)\leq \bar{w}(i,j)+(1-d)\cdot S$, where $\bar{w}(i,j)$ is already computed and *S* = ((*j* - *i* + 1) + (*k* - *i*))(*k* - *j*) / 2 is the number of unprocessed SNP pairs. Since *S* is largest for the longest region 〈*g*_*i*_, …, *g*_*n*_〉, we have $\text{max}\{\bar{w}(i,k)|k>j\}\leq \bar{w}(i,j)+(1-d)\cdot ((j-i+1)+(n-i))(n-j)/2$, and the estimated upper bound ${\bar{w}}_{\text{max}}(i,j)$ is defined as follows:

$${\bar{w}}_{\text{max}}(i,j)=\bar{w}(i,j)+(1-d)\cdot ((j-i+1)+(n-i))(n-j)/2.$$

The MIG ^+^ algorithm performs at most *λn*(*n* - 1) / 2 computations, where *λ*, 1 - *d* ≤ *λ* ≤ 1, depends on the data. The worst case of *λ* = 1 occurs only in the unlikely situation when a few very large blocks span an entire chromosome.

#### The MIG ^++^ algorithm

A limitation of the MIG ^+^ algorithm is its blindness about the unprocessed area to the right of the current SNP *g*_*j*_. Assuming *strong LD* for all SNP pairs in this area results in a conservative upper bound, ${\bar{w}}_{\text{max}}(i,j)$, for the region weights. An additional optimization step allows to obtain a more precise estimate of ${\bar{w}}_{\text{max}}(i,j)$ and further prunes unnecessary computations. The pseudocode of the modified algorithm, MIG ^++^, is given in Algorithm A.3 (Additional file 1).

The improved algorithm is an iterative procedure that, at each iteration, scans the chromosome from left to right and computes the weights only for a limited number of SNP pairs. For a SNP *g*_*j*_, the SNP pairs considered in an iteration are restricted to a window of size *win*: only the weights *w*(*j* - 1, *j*), …, *w*(*t*, *j*) are computed, where *t*= max({*b*, *j* - *win*}) and 1 ≤ *win* ≤ *n* (Figure 3, right panel). At each new iteration, the window size is increased by a number of SNPs equal to *win*. Therefore, the number of computed SNP-pair weights increases proportionally. This allows a more precise estimation of the upper bounds for the region weights with every new iteration.

By considering all SNP-pair weights computed in all previous iterations for the estimation of the upper bound, ${\bar{w}}_{\text{max}}(i,j)$, the algorithm requires linear time for each individual SNP pair to sum up all weights inside the corresponding region. We use a computationally cheaper constant-time solution, though it may lead to a less accurate estimation. Since $\bar{w}(i,k)\leq \bar{w}(i,j)+\bar{w}(1,k)-\bar{w}(1,j)$, we have $\max\{\bar{w}(i,k)|k>j\}\leq \bar{w}(i,j)+\max\{\bar{w}(1,k)|k>j\}-\bar{w}(1,j)$. An upper bound ${\bar{w}}_{\text{max}}(i,j)$ can then be computed as follows:

$${\bar{w}}_{\text{max}}(i,j)=\bar{w}(i,j)+\max\{\bar{w}(1,k)|k>j\}-\bar{w}(1,j).$$

$\max\{\bar{w}(1,k)|k>j\}$ is computed in linear time after every scan of the chromosome, whereas $\bar{w}(1,j)$ is computed in constant time. Thus, the computation of the upper bound ${\bar{w}}_{\text{max}}(i,j)$ for each individual SNP pair requires only constant time.

When *win* = *n*, MIG ^++^ is identical to MIG ^+^. When *win* = 1, the number of iterations becomes too large, introducing a significant computational burden. We propose to set *win* = ⌈(*n* - 1)(1 - *d*) / 2⌉, that corresponds to 1 - *d* percent of all SNP pairs, which is the minimal fraction of SNP pairs that must be considered before one can be sure that an *n*-SNP segment is not a haplotype block.

The MIG ^++^ performs at most *λn*(*n* - 1) / 2 computations, where *λ*, 1 - *d* ≤ *λ* ≤ 1, depends on the data. However, the value of *λ* obtained with the MIG ^++^ algorithm is expected to be always smaller than that from the MIG ^+^ algorithm, because of the more precise estimation of ${\bar{w}}_{\text{max}}(i,j)$.

### Alternative methods to estimate the *D*^′^ CI

A critical step of the Gabriel *et al.*[23] approach is the estimation of the *D*^′^ CI. In a genome-wide context, this calculation can be repeated hundreds of millions of times. In Haploview, the CIs are obtained by means of the likelihood-based procedure proposed by Wall and Pritchard [27], which requires from 100 to 1,000 iterations. This method can be replaced with a computationally cheaper solution, based on an approximated estimator of the *D*^′^ variance, as proposed by Zapata *et al*. [26]. This solution would make the whole block recognition algorithm significantly faster.

#### The wall and pritchard (WP) method

The true allele frequencies of each SNP are assumed to be equal to the observed allele frequencies. The likelihood of the data in the four-fold table obtained by crossing any SNP pair, conditional to the |*D*^′^| value, can be expressed as *l* = *P*(*data*∣|*D*^′^|). *l* is evaluated at each value of |*D*^′^| = 0.001 × *p*, with *p* = 0, 1, …, 1000. CL is defined as the largest value of |*D*^′^| such that $\overset{p-1}{\underset{i=0}{\sum }}l(i)/\overset{1000}{\underset{i=0}{\sum }}l(i)\leq \alpha$, where *α* is the significance level. Similarly, CU is defined as the smallest value of |*D*^′^| such that $\overset{1000}{\underset{i=p+1}{\sum }}l(i)/\overset{1000}{\underset{i=0}{\sum }}l(i)\leq \alpha$.

#### The approximate variance (AV) method

Consider two SNPs, *u* and *v*, with alleles {*u*_1_, *u*_2_} and {*v*_1_, *v*_2_}, respectively. Let $n_{u_{i}v_{j}}$ and $f_{u_{i}v_{j}}$ denote, respectively, the absolute and relative frequencies of the four possible haplotypes, *u*_*i*_*v*_*j*_(*i*, *j* ∈ {1, 2}), with $f_{u_{i}}$ and $f_{v_{j}}$ being the marginal frequencies of the two SNPs. In total, $N=\sum n_{u_{i}v_{j}}$ haplotypes are observed. Zapata et al. [26] showed that the variance of *D*^′^ can be approximated as follows:

$$\begin{array}{lll} \;V(D^{\prime })\approx & \left(\;\left(1-|D^{\prime }|\right)\times \left(N\cdot V(D)-|D^{\prime }|D_{\text{max}}\right)\right)\; & \;\\ & \times \left(\left(f_{u_{1}}f_{1}+f_{u_{2}}f_{2}-2|D|\right)\right)\; & \;\\ & \left(+\;\;|D^{\prime }|f_{3}(1-f_{3})\right)/\left(N\cdot D_{\text{max}}^{2}\right),\; & \; \end{array}$$

where $D^{\prime }\;=\;D/D_{\text{max}};D\;=\;f_{u_{1}v_{1}}-f_{u_{1}}f_{v_{1}}$; *D*_*max*_ is $\min\{\;f_{u_{1}}(1-f_{v_{1}}),(1-f_{u_{1}})f_{v_{1}}\}$ when *D* > 0 or $\;\min\{\;f_{u_{1}}f_{v_{1}},(1-f_{u_{1}})(1-f_{v_{1}})\}$ when *D* < 0; *f*_1_ is $f_{v_{1}}$ when *D*^′^ > 0 or $f_{v_{2}}$ when *D*^′^ < 0; *f*_2_ is $f_{v_{2}}$ when *D*^′^ > 0 or $f_{v_{1}}$ when *D*^′^ < 0; *f*_3_ is $f_{u_{1}v_{1}}$, $f_{u_{1}v_{2}}$, $f_{u_{2}v_{1}}$, and $f_{u_{2}v_{2}}$ when *D*_*max*_ is $f_{u_{1}}f_{v_{1}}$, $f_{u_{1}}f_{v_{2}}$, $f_{u_{2}}f_{v_{1}}$, and $f_{u_{2}}f_{v_{2}}$, respectively; and

$$\;V(D)\approx \left(\;f_{u_{1}}f_{u_{2}}f_{v_{1}}f_{v_{2}}+D\;(f_{u_{2}}-f_{u_{1}})(\;f_{v_{2}}-f_{v_{1}})-D^{2}\right)/\mathrm{N.}$$

When *D*^′^ = ±1, then *V*(*D*^′^) = 0. The 1 - *α* CI of *D*^′^ is equal to $D^{\prime }\pm Z_{\alpha /2}\sqrt{V(D^{\prime })}$, where *Z*_*α*/2_ is the 1 - *α* / 2 percentile of the standard normal distribution.

### Experimental evaluation

The experimental evaluation was based on the phased CEPH genotypes included in the HapMap phase II (HapMapII) [28] and the 1000 Genomes Project phase 1 release 3 (1000G) [29] databases. The HapMapII dataset included 2,543,857 SNPs from 120 haplotypes (60 individuals) and the 1000G dataset included 10,858,788 SNPs from 170 haplotypes (85 individuals).

To compare the new algorithms to the standard Haploview, in terms of runtime and memory usage, the ideal solution would have been that of randomly sampling regions with different characteristics from the HapMapII or 1000G datasets. However, the Haploview algorithm was so computationally expensive that it prohibited to consider a sufficiently large number of random regions and, therefore, to obtain a representative sample of all possible scenarios over the whole genome. For this reason, we selected the regions such that the most extreme scenarios, in terms of median SNP minor allele frequency (MAF) and median inter-SNP distance, were covered. To identify such representative regions, we performed the systematic scan of all SNPs in the genome using a sliding window of 1,000 SNPs, after removing chromosomal centromeres and the HLA region. For each sliding region, the median MAF and inter-SNP distance were recorded (Additional file 1, Figure A.1). All regions were then represented in a two-dimensional Euclidean space, where the normalized inter-SNP distance was plotted against the normalized median MAF (Additional file 1, Figure A.2). A total of nine regions were chosen for the experiments: the eight regions located on the outermost boundaries of the Euclidean space and the region closest to the center of the space. These regions represent scenarios with extreme and moderate median MAF and median inter-SNP distance. The procedure was repeated using larger sliding windows of 5,000 to 30,000 SNPs. If not stated otherwise, in the experimental results we report median values over the nine regions for every different window size.

The block partitions obtained with the WP and AV methods for *D*^′^ CI estimation were compared in terms of total number of blocks, median number of SNPs per block, proportion of SNPs clustered into blocks, and median within block haplotype diversity. Haplotype diversity [19,20] is defined as the ratio between the number of common haplotypes and the total number of haplotypes within a block. Common haplotypes are those occurring more than once. The haplotype diversity index ranges from 0 (complete diversity) to 1 (no diversity).

The three MIG algorithms were implemented in C++. To guarantee a fair comparison, the original Java implementation of the Haploview algorithm was rewritten in C++, too. By default, Haploview considers only SNP pairs within a maximal distance of 500Kbp. We removed this constraint because it could affect the block partitions of very wide regions. For the WP method, we set the number of likelihood estimation points to 1,000 (100 in the original Haploview implementation). We didn’t consider the population specific two-, three-, and four-marker rules, proposed by Gabriel *et al.*[23] when very short regions are processed, because they have no impact on the computational efficiency of the algorithms. All experiments were run on a machine with an Opteron 8356 Quad Core (2.3GHz) CPU.

### Genome-wide association study of rheumatoid arthritis

We applied our haplotype block partitioning algorithm to the genome-wide association study of the North American Rheumatoid Arthritis Consortium (NARAC) dataset. Data consisted of 868 cases and 1,194 controls. The samples were genotyped at 544,917 autosomal and sex chromosome SNPs. Quality check was performed with PLINK 1.07 [25]: we excluded 5,422 SNPs with a call rate of <90*%*, 11,327 SNPs with a minor allele frequency of <0.001, and 898 SNPs because of significant deviation from Hardy-Weinberg equilibrium in controls (*p-value* ≤ 10^-6^). No samples were excluded because of low call rate (<90*%*); 2 cases and 5 controls were removed because of sex mismatch; 1 case and 8 controls were additionally excluded after population stratification test based on principal component analysis performed with EIGENSOFT 5.0.1 [30]. After the quality control, 514,539 autosomal SNPs and 2,046 samples were available for analyses.

Haplotypes were phased using SHAPEIT version 2 [31]. To achieve good accuracy, we set 400 conditioning states per SNP. Recombination rates were taken from HapMap phase II build 36 and effective population size was set to 11,418 (as suggested for CEU populations). The estimated haplotypes were submitted to MIG ^++^ and processed with the WP and AV methods. We obtained 98,979 WP blocks, covering 445,832 SNPs, with 68,707 singleton SNPs outside of any block. The AV method identified 97,816 blocks, covering 446,170 SNPs, and 68,369 singleton SNPs.

The genome-wide association scan was based on a logistic regression model adjusted for sex and the top 10 eigenvectors obtained from EIGENSOFT 5.0.1 [30]. The association between disease status and individual SNPs or haplotype blocks was tested with a likelihood ratio test using PLINK 1.0.7 [25] with the logistic-genotypic and omnibus options, respectively. Within each block, haplotypes with frequency of <0.01 were collapsed together to preserve power. Singleton SNPs outside blocks were treated as in the SNP-based analysis, therefore producing analogous results. Genomic control (GC) correction was applied to both SNP- and block-based GWAS results. Bonferroni-corrected significance thresholds were set to 2.98 × 10^-7^ for analysis based on the WP block partition (i.e. 0.05 divided by the sum of 98,979 WP blocks and 68,707 singleton SNPs), 3.01 × 10^-7^ for the analysis based on the AV method (i.e. 0.05 divided by 97,816 AV blocks plus 68,369 singleton SNPs), and 9.17 × 10^-8^ (i.e. 0.05 / 514,539) for the individual SNP analysis.

## Results

### Runtime and memory with the WP method

**Figure 4:**
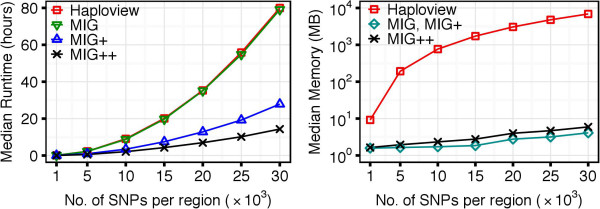
**Performance of the algorithms with the WP method, when applied to the 1000G dataset.** MIG ^+^ and MIG ^++^ show substantially better runtime than MIG and Haploview. All versions of the MIG algorithm significantly outperforms Haploview in memory usage and, therefore, are able to handle densely genotyped and genome-wide data.

Figure 4 shows runtime and memory performance of Haploview and the three MIG algorithms based on the WP method, when applied to the 1000G dataset. Since both Haploview and MIG perform *n*(*n* - 1) / 2 computations, it was expected to see identical runtime: both of them took 80 hours to process regions of 30,000 SNPs. However, MIG used three orders of magnitude less memory than Haploview (3 MB vs. 7 GB). The runtime was significantly reduced with MIG ^+^ (27 hours) and even further with MIG ^++^ (14 hours). The runtime difference between algorithms increased with the region size (number of SNPs). Memory usage was identical for MIG and MIG ^+^, whereas MIG ^++^ required slightly more memory to store the computational status between iterative region scans. Similar results were obtained on the HapMapII dataset (results not shown).

**Figure 5:**
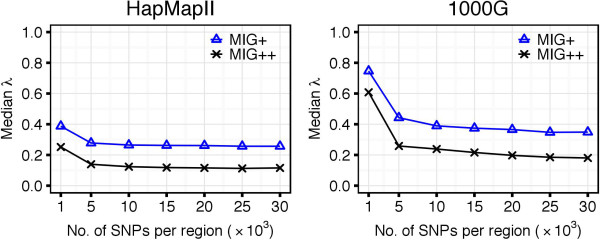
**Values of the λ coefficient for MIG**^**+ **^**and MIG**^**+ **^**with the WP method.** MIG ^+^ omits more SNP-pair weight computations than MIG ^+^, which is reflected by the smaller *λ* coefficient.

**Figure 6:**
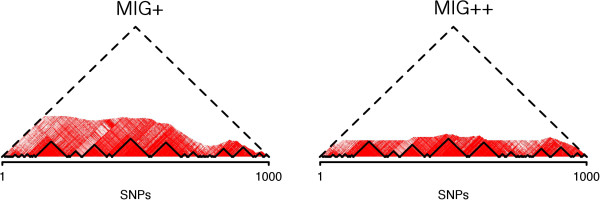
**LD heatmap of region chr20:31,767,872-33,700,401, which contains 1,000 polymorphic SNPs in the HapMapII dataset.** The area under the dashed line corresponds to all possible SNP pairs. The computed SNP-pair weights are indicated in red, with higher red intensity corresponding to higher |*D*^′^| values. The non-colored area corresponds to the SNP pairs omitted from computations. The solid line indicates the final haplotype block partition. It can be observed that, while the final block partition is identical for the two algorithms, more computations have been omitted by MIG ^+^ than MIG ^+^.

The MIG ^++^ omitted more unnecessary computations than MIG ^+^, which is reflected by the smaller *λ* coefficient in both HapMapII and 1000G datasets (Figure 5). The *λ* values decreased with increasing number of SNPs in the region. When increasing the region size, after a rapid decline for small regions, *λ* reached stable values with both MIG ^+^ and MIG ^++^ algorithms and in both datasets. This behavior relates to the LD decay with distance. In regions of 30,000 SNPs in the 1000G dataset, the MIG ^++^ algorithm was able to omit ∼80*%* of the calculations (*λ* ∼0.20), while MIG ^+^ could omit ∼60*%* of the calculations (*λ*∼0.40). An example of the reduction of the number of calculations is given in Figure 6, where MIG ^+^ and MIG ^++^ are compared to Haploview, which is represented by the entire triangle.

### Runtime and memory with the AV method

**Figure 7:**
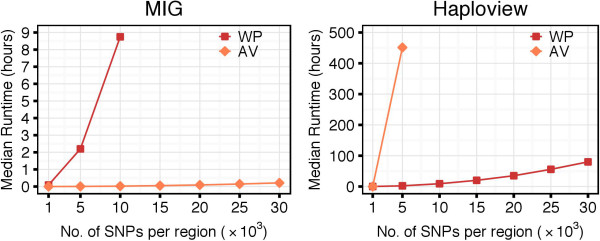
**Impact of the WP and AV methods on runtime, when applied to the 1000G dataset.** The AV method had negative impact on Haploview runtime: the processing was significantly slower compared to the WP method. However, when combined with MIG, the AV method reduced the computational time from hours to seconds, compared to the WP method.

When we introduced the AV method to estimate the *D*^′^ CI, we observed a drastic reduction of the computational time of the MIG algorithm. With the AV approach, the median runtime needed to analyze sequences of 10,000 SNPs in the 1000G dataset was of 2 minutes. The same analysis took a median of 8.7 hours with the WP method (Figure 7, left panel). Proportional time reduction was observed for MIG ^+^ and MIG ^++^. Similar results were obtained in the HapMapII dataset (results not shown).

**Figure 8:**
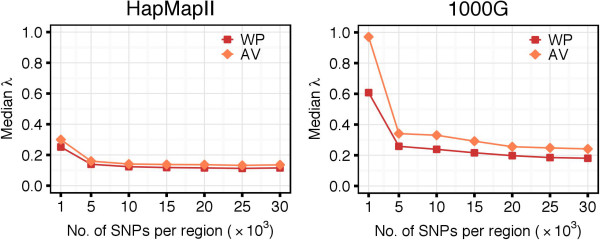
**Values of the λ coefficient for MIG**^**+**^**: comparison between WP and AV methods.** When using the AV method, more SNP-pair weights were computed and, therefore, the *λ* coefficient was greater compared to that from the WP method. However, for its higher the runtime efficiency, the AV method still allowed to significantly decrease the computational time compared to the WP method.

We observed that the introduction of the AV method caused a slight increase of the *λ* coefficient (Figure 8). This is because, with the AV method, more SNP pairs are classified to be in *strong LD*. This causes an increase of the number of possible configurations to be checked and results in a larger set of candidate haplotype blocks. With the AV method, the MIG algorithms identified tens of millions of candidate haplotype blocks (Additional file 1, Figure A.3). The number of candidate blocks was even larger when the AV method was applied directly to Haploview, where candidate blocks need to satisfy only Definition 1 (a). This significantly larger number of candidate blocks explains the increase in runtime of Haploview when using the AV method: for regions of 5,000 SNPs in the 1000G dataset, the median runtime was of 451 hours with the AV against the 2 hours with the WP method (Figure 7, right panel).

### Block Partitions with the WP and AV Methods

**Figure 9:**
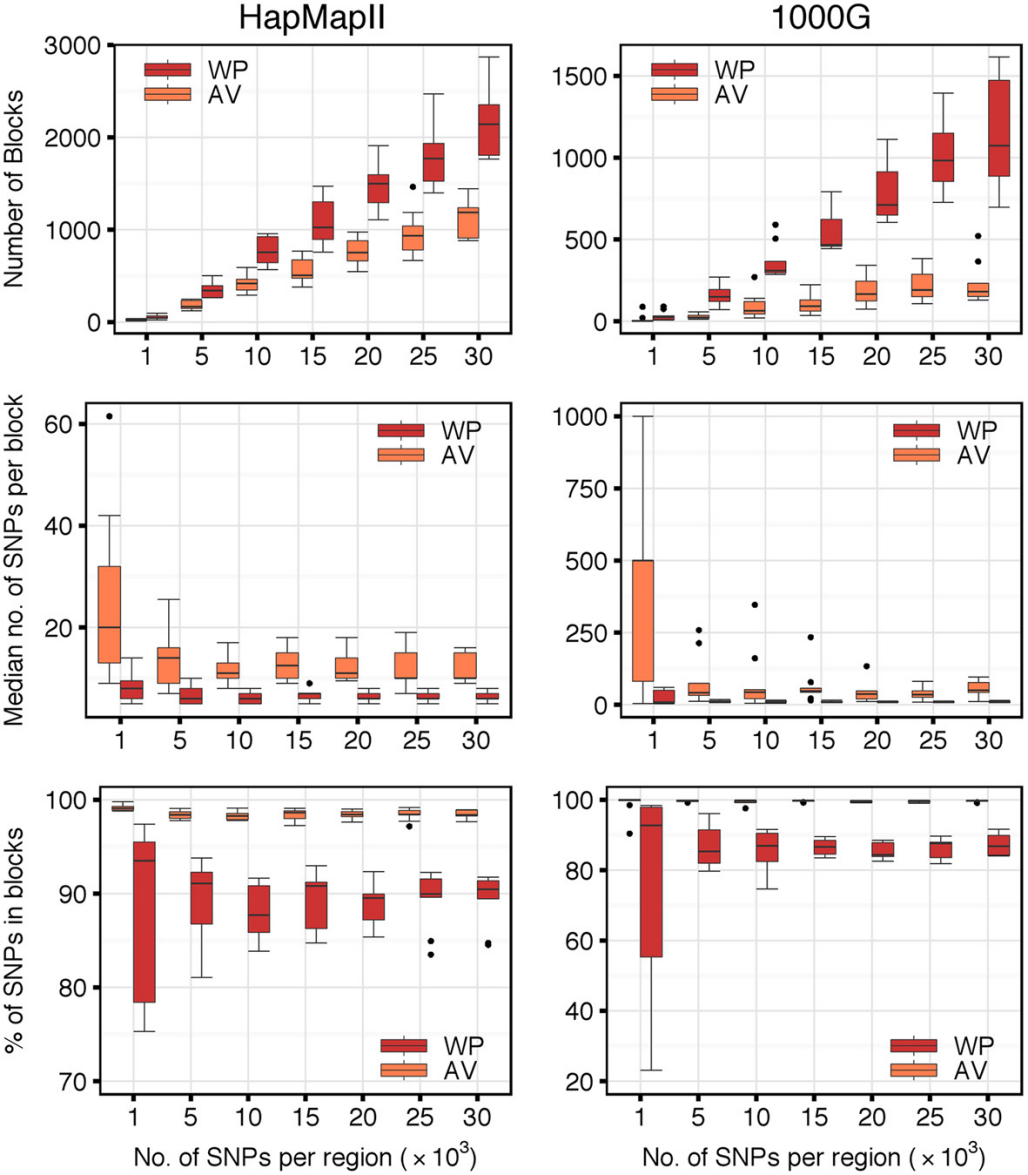
**Haplotype block characteristics of WP and AV methods.** Top panels show the number of haplotype blocks per region, middle panels show the median number of SNPs per block, and bottom panels show the overall percentage of SNPs in haplotype blocks.

The characteristics of the different block partitions obtained with the WP and AV methods are summarized in Figure 9. The AV method produced a smaller number of blocks than the WP method (top panels). The median number of blocks per region increased along with the number of SNPs, and it increased faster for the WP compared to the AV method. Considering the median number of SNPs per block, the AV method produced larger blocks than the WP method (middle panels). For very short regions (e.g., 1,000 SNPs) both methods generally induced larger blocks. This is because such small regions might be completely covered by a single or very few haplotype blocks. The median number of SNPs per block decreased along with the increase of the length of the region considered. Overall, the AV method assigned a higher percentage of SNPs to blocks compared to the WP method, which left more singleton SNPs outside of any block (bottom panels). In the analysis of the HapMapII dataset, 98.4*%* of the SNPs were clustered within blocks with the AV method and 90.5*%* with the WP method. In the analysis of the 1000G dataset, the percentages were of 99.7 and 86.8, respectively.

**Figure 10:**
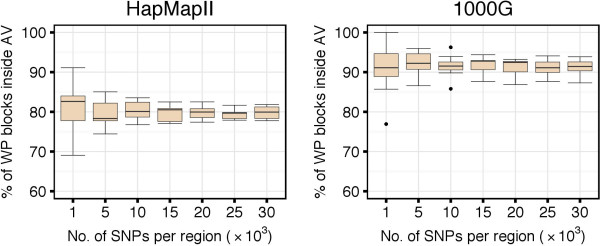
**Number of blocks detected with the WP method that are completely inside blocks detected with the AV method.** Independently of the region size, from 80% to 90% of haplotype blocks detected with the WP method were completely inside haplotype blocks detected with the AV method.

We observed that 100% of the blocks identified by the WP method were overlapping blocks identified by the AV method. More specifically, 80% to 90% of the blocks based on the WP method were completely included within blocks based on the AV method (Figure 10). The remaining 10% to 20% of WP blocks whose borders were crossing borders of AV blocks, could be entirely attributed to the selection mechanism in the step (2) of the algorithm, when larger candidate blocks are prioritized over the shorter ones. In fact, when, instead of looking at the final block partition, we focused on the intermediate set of candidate blocks before the final pruning, we observed that 100% of the candidates from the WP method were entirely included within the candidate blocks from the AV method.

**Figure 11:**
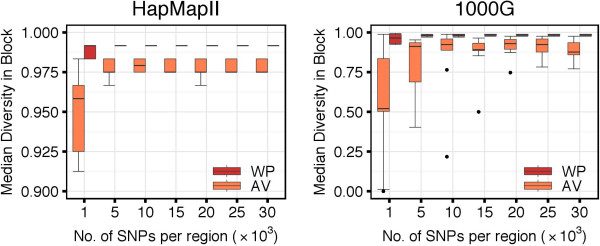
**Within-block haplotype diversity with WP and AV methods.** AV method-based haplotype blocks showed higher haplotype diversity compared to WP method-based haplotype blocks. For regions with more than 10,000 SNPs, the median haplotype diversity within blocks was low (>0.8).

Consistently with the findings of larger AV blocks, we observed a generally higher haplotype diversity in the partitions obtained with the AV method compared to the WP method (Figure 11). For instance, when considering regions of 30,000 SNPs in the 1000G dataset, we observed median within-block haplotype diversity indices of 0.876 and 0.982 with the AV and WP methods, respectively. Slightly higher diversity indices were observed in the HapMapII dataset: 0.975 and 0.992 for the AV and WP methods, respectively. The within-block diversity was more variable in short than in long regions because, as observed above, when regions are too small, then it might be difficult to identify more than one block.

### Whole genome haplotype block recognition

**Figure 12:**
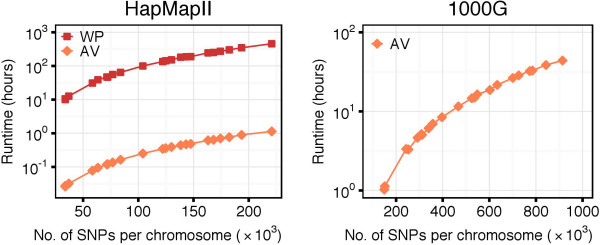
**Runtime of the MIG**^**++ **^**algorithm on whole-genome data.**

The linear memory complexity and the significant reduction of the number of computations allowed us to run MIG ^++^ on a genome-wide scale. We could run MIG ^++^ on the full HapMapII dataset using both the WP and AV methods for *D*^′^ CI derivation. Using the more efficient AV method, we were also able to run a genome-wide haplotype block partition of the complete 1000G dataset. The runtime for the two datasets is shown in Figure 12. For HapMapII, the maximal runtime was of 1 hour when using the AV method and of 457 hours when using the WP method. In both cases, the maximal runtime was observed for chromosome 2, which contained 220,833 SNPs. The median *λ* value across all chromosomes was 0.129 (min = 0.125, max = 0.133) for the AV method and 0.103 (min = 0.099, max = 0.110) for the WP method. For the 1000G dataset, the maximal runtime using the AV method was of 44 hours on chromosome 2, which contained 913,923 SNPs. The median *λ* value across all chromosomes was 0.216 (min = 0.206, max = 0.224). The maximal memory usage was very low and didn’t exceed 151 MB and 3.6 GB for the HapMapII and 1000G datasets, respectively.

**Figure 13:**
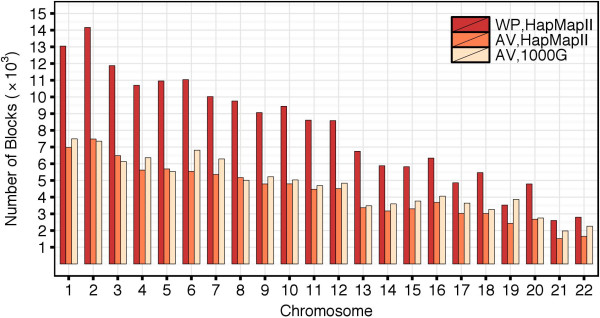
**Number of haplotype blocks in the HapMapII and 1000G datasets when the *D***^***′ ***^**CIs are estimated with the WP and AV methods.**

Figure 13 shows the number of haplotype blocks per chromosome. In the HapMapII dataset, the largest number of blocks occurred in chromosome 2: 14,164 blocks with the WP method and 7,482 blocks with the AV method. The number of blocks detected with the WP method was always exceeding the number of blocks detected with the AV method. For some chromosomes, the partitions obtained by the WP method contained almost twice as many blocks as the partitions obtained by the AV method. When using the AV method, we detected a very similar number of blocks in the HapMapII and 1000G datasets. Across all chromosomes, 100% of the blocks detected with the WP method were overlapping blocks detected with the AV method. The median percentage of the WP blocks completely covered by the AV blocks was 0.797 (min = 0.773, max = 0.813).

The characteristics of the whole-genome block partitions obtained with the AV and WP methods are summarized in Table 1. The results were similar to the experiments on smaller regions. In the HapMapII dataset, fewer and larger blocks were detected with the AV method than with the WP method. With the AV method, a higher percentage of SNPs was assigned to blocks and the within-block haplotype diversity index was slightly smaller. However, the haplotype diversity was close to one for both methods, indicating that in both cases the number of possible haplotypes should be very limited. When applying the AV-based MIG ^++^ algorithm to the 1000G dataset, we observed a higher percentage of SNPs in blocks and a slightly smaller diversity index, which is explained by the higher number of SNPs per block.

**Table 1:** Characteristics of the whole-genome haplotype block partitions obtained with the WP and AV methods

	**WP**			**AV**		
	**Median**	**Min**	**Max**	**Median**	**Min**	**Max**
	**HapMapII**					
No. of SNPs per Block	6	5	7	12	7	14
% of SNPs in Blocks	89.6	85.8	91.4	98.5	97.2	98.8
Diversity in Block	0.992	0.992	0.992	0.975	0.975	0.983
	**1000G**					
No. of SNPs per Block	–	–	–	25	13	34
% of SNPs in Blocks	–	–	–	99.4	99.1	99.6
Diversity in Block	–	–	–	0.944	0.929	0.971

For both HapMapII and 1000G datasets, the largest blocks were located over the chromosomal centromeres and spanned tens of millions of base-pairs (bp) (Additional file 1, Figure A.4). Some of these very large blocks were characterized by very low and irregular SNP density. After filtering out these exceptionally large blocks, the largest block identified by the WP-based MIG ^++^ algorithm in the HapMapII dataset was located in chromosome 1, it was 1,017,844 bp long and included 398 SNPs. When using the AV method, the largest block was located in chromosome 12, it was 1,190,412 bp long and included 335 SNPs. In the 1000G dataset, the largest block detected by the AV-based MIG ^++^ was located in chromosome 1, it was 1,361,781 bp long and included 2,896 SNPs.

### Genome-wide association study of rheumatoid arthritis

After the GWAS, we observed a genomic inflation factor *λ* of 1.015 for the SNP-based analysis, 1.082 for the AV block-based analysis, and 1.077 for the WP block-based analysis. After GC correction, in the SNP-based analysis, 116 SNPs were genome-wide significant. Of them, 106 were located inside 25 AV blocks and 110 inside 27 WP blocks. From the AV and WP block-based analyses, we observed 29 and 33 genome-wide significant blocks, respectively. Twenty-three of such blocks were the same between the two methods. The results from the SNP- and block-based analyses are compared in Table 2. The first part of the table shows the 20 genome-wide significant loci detected by both SNP- and block-based analyses. In most cases, the AV and WP methods brought to identical results. One exception was the 4^*th*^ locus, where two adjacent AV blocks including 6 and 14 SNPs, respectively, corresponded to two adjacent WP blocks of 7 and 13 SNPs, respectively. That is, one SNP shifted from one block to another. In terms of significance, results were practically unchanged. A second exception was locus 13, were a block was detected only with the WP but not with the AV method. The last two exceptions were loci number 15 and 19. In both cases, an AV block was split into two WP blocks. The second part of Table 2 shows a number of loci that wouldn’t have been detected with a SNP-based GWAS, but were uncovered by at least one of the two block partition methods. The AV and WP methods produced similar results. We didn’t observe any clear advantage of one method compared to the other. The last section of the table shows that there was a small number of loci uncovered only by the SNP-based analysis. For these loci, the p-values from the block-based analyses were often close to the significance level, with the exception of the last two loci.

**Table 2:** Results from the rheumatoid arthritis GWAS: comparison between AV and WP haplotype blocks and single-SNP analyses

		**Block**				**Top SNP**	
**Locus**	**Partition method**	**Chr:start-end**	**# sign. SNPs**	**# SNPs**	**P-value**	**Name**	**P-value**
Genome-wide significant blocks that include genome-wide significant SNPs							
1	AV & WP	6:32,055,439-32,182,782	2	15	**2.71 × 10**^**-12**^	rs2239689	**1.55 × 10**^**-8**^
2	AV & WP	6:32,204,222-32,259,421	1	14	**1.32 × 10**^**-16**^	rs3134943	**5.41 × 10**^**-8**^
3	AV & WP	6:32,317,005-32,319,063	2	3	**1.25 × 10**^**-8**^	rs412657	**2.17 × 10**^**-10**^
4	AV	6:32,323,166-32,328,663	4	6	**1.99 × 10**^**-15**^	rs9267992	**1.14 × 10**^**-11**^
	AV	6:32,331,236-32,390,832	4	14	**2.34 × 10**^**-33**^	rs6910071	**1.92 × 10**^**-32**^
	WP	6:32,323,166-32,331,236	5	7	**1.32 × 10**^**-17**^	rs3130320	**7.50 × 10**^**-17**^
	WP	6:32,332,366-32,390,832	3	13	**1.38 × 10**^**-33**^	rs6910071	**1.92 × 10**^**-32**^
5	AV & WP	6:32,397,296-32,445,664	13	24	**3.00 × 10**^**-24**^	rs547077	**2.60 × 10**^**-18**^
6	AV & WP	6:32,454,772-32,471,794	7	9	**4.88 × 10**^**-27**^	rs3817973	**5.48 × 10**^**-26**^
7	AV & WP	6:32,474,399-32,476,065	1	3	**2.00 × 10**^**-23**^	rs3817963	**8.55 × 10**^**-23**^
8	AV & WP	6:32,477,466-32,481,676	1	3	**7.75 × 10**^**-21**^	rs3806156	**9.43 × 10**^**-14**^
9	AV & WP	6:32,483,951-32,491,086	5	8	**7.93 × 10**^**-38**^	rs3763312	**7.00 × 10**^**-34**^
10	AV & WP	6:32,491,201-32,509,057	6	8	**5.10 × 10**^**-41**^	rs2395163	**6.14 × 10**^**-37**^
11	AV & WP	6:32,509,195-32,514,320	6	8	**1.80 × 10**^**-37**^	rs2395175	**1.83 × 10**^**-39**^
12	AV & WP	6:32,519,501-32,521,295	3	3	**1.79 × 10**^**-27**^	rs7192	**2.64 × 10**^**-26**^
13	WP	6:32,522,251-32,535,767	2	2	**1.10 × 10**^**-28**^	rs9268832	**4.07 × 10**^-25^
14	AV & WP	6:32,541,145-32,713,862	7	12	**2.23 × 10**^**-47**^	rs660895	**2.60 × 10**^**-45**^
15	AV	6:32,760,295-32,766,057	2	5	**4.99 × 10**^**-26**^	rs9275184	**1.49 × 10**^**-18**^
	WP	6:32,735,692-32,762,692	3	4	**8.33 × 10**^**-35**^	rs9275184	**1.49 × 10**^**-18**^
	WP	6:32,763,196-32,766,057	1	3	**3.33 × 10**^**-23**^	rs7774434	**5.43 × 10**^**-10**^
16	AV & WP	6:32,766,602-32,785,130	26	37	**1.71 × 10**^**-40**^	rs9275224	**1.31 × 10**^**-37**^
17	AV & WP	6:32,786,977-32,790,115	6	10	**9.32 × 10**^**-50**^	rs9275595	**1.97 × 10**^**-28**^
18	AV & WP	6:32,792,235-32,827,644	2	23	**2.94 × 10**^**-19**^	rs3916765	**2.72 × 10**^**-9**^
19	AV	6:32.912,776-32,912,887	1	2	**1.46 × 10**^**-8**^	rs3819721	**5.74 × 10**^**-9**^
	WP	6:32,912,392-32,912,776	1	2	**9.89 × 10**^**-10**^	rs3819721	**5.74 × 10**^**-9**^
	WP	6:32,912,887-32,912,912	0	2	**7.18 × 10**^**-11**^	rs241425	7.53 × 10^-4^
20	AV & WP	9:81,662,684-81,666,969	1	2	**2.72 × 10**^**-8**^	rs7854383	**3.23 × 10**^**-8**^
Genome-wide significant blocks with no corresponding genome-wide significant SNPs							
21	AV & WP	2:219,728,763-219,836,597	0	17	**1.57 × 10**^**-7**^	rs1052483	2.02 × 10^-4^
22	AV	6:31,723,146-31,726,740	0	3	3.17 × 10^-7^	rs3130050	5.27 × 10^-5^
	WP	6:31,723,146-31,726,740	0	3	**2.98 × 10**^**-7**^	rs3130050	5.27 × 10^-5^
23	AV & WP	6:31,728,499-31,777,475	0	10	**4.11 × 10**^**-8**^	rs2280800	1.28 × 10^-4^
24	AV	6:31,910,520-31,953,964	0	10	**8.66 × 10**^**-8**^	rs9267658	1.22 × 10^-5^
	WP	6:31,885,925-31,945,256	0	8	1.08 × 10^-5^	rs2075800	3.58 × 10^-5^
	WP	6:31,946,420-31,953,964	0	5	1.73 × 10^-6^	rs9267658	1.22 × 10^-5^
25	AV	6:31,958,311-31,959,213	0	2	6.00 × 10^-1^	rs652888	1.48 × 10^-2^
	AV	6:31,968,316-32,026,839	0	10	1.53 × 10^-5^	rs1042663	7.47 × 10^-7^
	WP	6:31,959,213-32,026,839	0	11	**4.59 × 10**^**-8**^	rs1042663	7.47 × 10^-7^
26	AV & WP	6:32,027,809-32,038,441	0	5	**1.43 × 10**^**-9**^	rs437179	2.20 × 10^-5^
27	AV & WP	6:32,262,976-32,263,559	0	2	**8.89 × 10**^**-9**^	rs204994	1.83 × 10^-4^
28	AV & WP	6:32,296,361-32,298,006	0	4	**1.47 × 10**^**-10**^	rs3132946	1.51 × 10^-7^
29	AV & WP	6:32,300,538-32,303,337	0	5	**6.87 × 10**^**-10**^	rs499691	5.62 × 10^-4^
30	AV	6:32,870,369-32,889,502	0	12	**4.33 × 10**^**-10**^	rs7767167	2.61 × 10^-4^
	WP	6:32,871,088-32,889,502	0	11	**3.77 × 10**^**-10**^	rs7767167	2.61 × 10^-4^
31	AV	6:32,912,776-32,912,887	1	2	**1.46 × 10**^**-8**^	rs3819721	**5.74 × 10**^**-9**^
	WP	6:32,912,392-32,912,776	1	2	**9.89 × 10**^**-10**^	rs3819721	**5.74 × 10**^**-9**^
	WP	6:32,912,887-32,912,912	0	2	**7.18 × 10**^**-11**^	rs241425	7.53 × 10^-4^
32	AV & WP	6:33,012,959-33,069,082	0	21	**8.10 × 10**^**-8**^	rs3135034	5.13 × 10^-5^
Genome-wide significant SNPs with no corresponding genome-wide significant blocks							
33	AV & WP	1:18,189,820-18,200,270	1	8	1.81 × 10^-5^	rs16861613	**5.08 × 10**^**-8**^
34	AV & WP	6:32,307,122-32,313,088	2	5	2.04 × 10^-6^	rs9267873	**3.98 × 10**^**-8**^
35	AV	10:112,614,407-112,822,215	1	23	5.73 × 10^-7^	rs3750619	**8.29 × 10**^**-10**^
	WP	10:112,614,407-112,749,598	0	18	3.37 × 10^-7^	rs3750619	**8.29 × 10**^**-10**^
	WP	10:112,754,584-112,822,215	0	5	1.89 × 10^-1^	rs10787298	1.10 × 10^-1^
36	AV & WP	17:34,570,514-34,575,487	1	5	1.40 × 10^-1^	rs593772	**5.50 × 10**^**-9**^
37	AV & WP	17:63,271,780-63,418,511	1	8	3.26 × 10^-1^	rs7502707	**2.02 × 10**^**-8**^

## Discussion

We propose an algorithm for haplotype block partitioning, termed MIG ^++^, which represents a scalable implementation of the Haploview algorithm and produces the same results in a much shorter time and using a substantially smaller amount of main memory. MIG ^++^ can process large DNA regions using only a handful of megabytes of main memory. In such situations, Haploview would require gigabytes. In terms of runtime, the MIG ^++^ is several times faster than Haploview. We also demonstrated that more than 80% of calculations were not necessary for the purpose of block recognition and could be omitted, thus achieving a higher efficiency. The improved performance of the algorithm makes it possible to process very large chromosomal segments. When the approximated variance estimator, proposed by Zapata *et al.*[26], is used to estimate the *D*^′^ CI, the MIG ^++^ can be applied genome-wide and process high density datasets, such as the 1000G, in a very short time.

With its very small memory requirements, the MIG ^++^ can process any number of SNPs. This allowed us to avoid Haploview’s restrictions on the maximal haplotype block length (the default limitation is 500Kbp) and to consider the LD between SNPs at any distance. Our whole-genome experiments showed that the haplotype blocks, based on the Gabriel *et al.*[23] definition, can span more than 500Kbp and can extend over several millions of base pairs. This empirical result suggests that limiting the maximal block length may alter the block partition. The alteration can be substantial because the algorithm prioritizes the largest blocks. The smallest blocks are retained only when they do not overlap with the largest ones. For this reason, to constrain the block length within pre-specified limits may induce a cascade of effects and may affect the final partition of very large segments. This is relevant, for example, when assessing the LD pattern of loci selected from GWAS, with the aim of identifying genes related to the lead SNP. In such cases, different partitions could imply different genes to be selected for follow-up.

With the MIG ^++^ algorithm, we were able to run a haplotype block recognition of the entire HapMapII dataset. However, it still required an unacceptably long time to apply the algorithm to larger and denser genomes, such as the 1000G dataset. This limitation is due to the use of the Wall and Pritchard [27] method, which models the |*D*^′^| likelihood and derives the |*D*^′^| CIs using an iterative procedure. In contrast, if the *D*^′^ variance is estimated with the approximated formula suggested by Zapata *et al.*[26], it is possible to derive the *D*^′^ CI with a single mathematical calculation. Thanks to this computationally less demanding solution, we could perform a complete block recognition of the HapMapII dataset in 1 hour and to process the entire 1000G dataset in 44 hours. To the best of our knowledge, this is the first time that such a marker-dense genome has been partitioned with a threshold-free approach. Previously, block partition of the whole genome could only be achieved by dividing chromosomes into small chunks or by restricting computations using sliding window approaches. Such choices may introduce artificial breaks to the real haplotype structure.

It is important to note that the block partition obtained with an algorithm based on the AV method is not fully equivalent to a partition obtained based on the WP method. For large sample sizes and for common variants, the estimated variance of the *D*^′^ statistic is going to be similar, whichever method is used. However, when crossing a common with a rare SNP, it often happens that one of the four possible haplotypes is not present in the sample. In such situations, it is very likely that the *D*^′^ CI shrinks to 1 because the approximated variance is zero. In this way, the SNP pair is systematically classified as a *strong LD* pair. As a result, SNPs with rare alleles are easily grouped together into very large blocks, boosting the region coverage and the median number of SNPs per block. The WP method is less sensitive to extreme *D*^′^ values, and the resulting blocks are generally shorter. However, we observed that most (80%) of the haplotype blocks obtained with the WP method were contained within the larger blocks obtained with the AV method. That is, the use of the AV method produced a coarser partition, where AV blocks entirely contained one or more WP blocks. For this reason, the AV blocks showed a higher haplotype diversity, in the terms described by Patil *et al.*[19] and Zhang *et al.*[20], than the WP blocks.

To provide an application of a whole-genome haplotype block partition, we analyzed the data from the North American Rheumatoid Arthritis Consortium (NARAC) dataset using both block partitions: the one obtained with the standard WP method and the one obtained with the AV approach. As observed in previous studies [32,33], the GWAS results were dominated by the HLA locus on chromosome 6. However, other loci were identified in other chromosomes. For what concerns the two block partition methods, the results were very similar, suggesting that the AV approach might be a convenient way to run a fast recognition of the haplotype blocks. However, we recognize that ours was an empirical application based on half a million genotyped SNPs. Results might be different in a larger context, such as that of a GWAS based on the 1000 Genomes dataset, where the number of AV blocks is expected to be much smaller than the number of WP blocks, and the AV blocks are expected to be much larger than the WP ones. Our empirical analysis of the NARAC data also confirmed previous observations that SNP- and block-based analyses are complementary to each other [32,34]. In fact, in our analysis some loci were identified only by the single-SNP analysis, other loci were identified only by the haplotype-block analysis, and others by both methods. Thus, genome-wide haplotype association scans are not in competition with standard GWAS. Genome-wide haplotype association scans should be considered as complementary tools that may help to identify loci that could be overlooked by methods based on single-SNP analysis. We also observed that haplotype blocks may simplify gene annotation. While only one gene, the HLA-DRA [35], which was reported by previous GWASs, was directly implied by a genome-wide significant SNP, four additional previously reported genes were implied by genome-wide significant blocks: the APOM, HLA-DQA1, HLA-DRB1, and HLA-DQA2 genes [35].

## Conclusions

**Figure 14:**
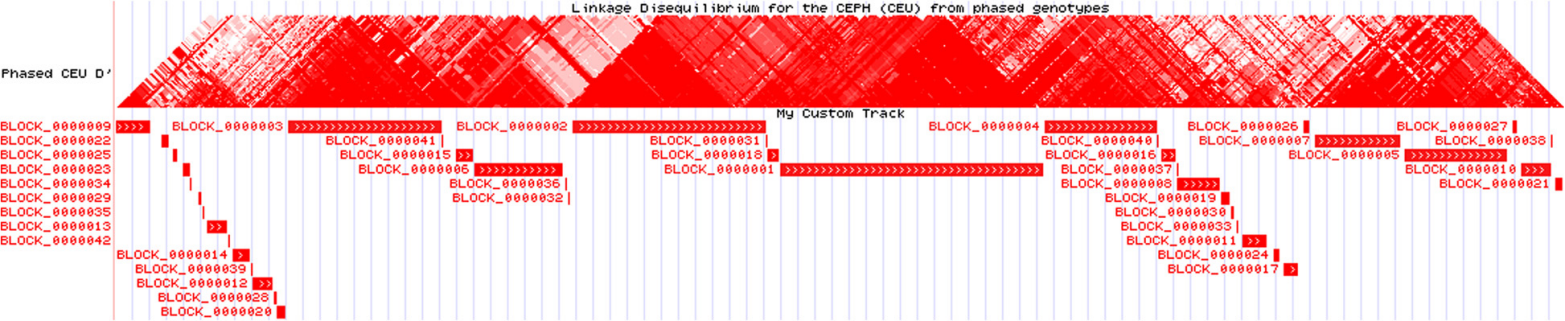
**Visualization of the LD-based haplotype block structure of a chromosome region using the UCSC genome browser **[17]**.** Top panel: the pairwise LD values in chr20:31,767,872-33,700,401 calculated by the UCSC genome browser using Haploview for SNPs within 250 kb. Bottom panel: LD-based haplotype blocks obtained with MIG ^++^ using LD between SNPs at any distance.

We have provided an efficient and scalable haplotype block recognition algorithm, termed MIG ^++^, which improves the well-known Haploview algorithm by reducing memory complexity from quadratic to linear and by omitting approximately 80% of unnecessary computations. The improved algorithm was able to efficiently process dense genomic segments of any size. When applied to individual-level data, where genotypes are available, the MIG ^++^ efficiency can be exploited to set up haplotype-based (genome-wide) association scans that could account for the correct underlying haplotype distribution. This seems to be especially relevant when rarer variants are involved. If ran on summary results from GWAS, the MIG ^++^ could help identify biologically plausible scenarios for SNP-set analysis and it could support a more correct annotation of genes surrounding variants of interest. From a population-genetic point of view, the method could facilitate the comparison of human genomes across different ethnicities, helping to highlight structural differences. Finally, the algorithm opens up the possibility to integrate genome-wide LD-based haplotype block structure into visual assessment tools, thus improving the interpretation of already available, but incomplete, LD heatmaps (Figure 14).

The MIG algorithms are available in the *LDExplorer* R package at http://www.eurac.edu/LDExplorer together with usage instructions and examples. Further improvements will include application of parallel computation techniques to MIG ^++^ in order to further speed up the processing while keeping memory requirements low.

## Competing interests

The authors declare that they have no competing interests.

## Authors’ contributions

DT developed the MIG algorithms, implemented all algorithms in C++, designed and performed all experiments, and drafted the manuscript. JG supervised the project and revised the manuscript. CP supervised the project, planned the experiments, and revised the manuscript. The authors read and approved the manuscript.

## Supplementary Material

Additional file 1 **Appendix.** Includes pseudocode for the MIG algorithms, illustrations of chromosomal regions sampling procedure, and figures illustrating various haplotype block properties. Click here for file
