# Supplementary material for: Efficient haplotype block recognition of very long and dense genetic sequences
Source: BMC Bioinformatics. 2014 Jan 14;15:10. doi: 10.1186/1471-2105-15-10 (PMC3898000; doi:10.1186/1471-2105-15-10)
Supplement: Additional file 1 — Appendix. Includes pseudocode for the MIG algorithms, illustrations of chromosomal regions sampling procedure, and figures illustrating various haplotype block properties. [file 1471-2105-15-10-S1.pdf]

APPENDIX

Algorithm A.1: MIG

```
Data: C = ⟨g_1, ... g_n⟩
Result: H = ∅
W ← ⟨0, ..., 0⟩ ;
for j = 2 to n do
    s ← 0;
    for  i = j − 1 downto 1 do
        w ← w(i, j);
        s ← s + w;
        W[i] ← W[i] + s;
        if w = 1 − d and W[i] ≥ 0 then
            H ← H ∪ ⟨g_i, ..., g_j⟩;
return H;
```

Algorithm A.2: MIG$^+$

```
Data: C = ⟨g_1, ... g_n⟩
Result: H = ∅
W ← ⟨0, ..., 0⟩;
new_b ← 1;
for j = 2 to n do
    s ← 0;
    b ← new_b;
    new_b ← j;
    for  i = j − 1 downto b do
        w ← w(i, j);
        s ← s + w;
        W[i] ← W[i] + s;
        if w = 1 − d and W[i] ≥ 0 then
            H ← H ∪ ⟨g_i, ..., g_j⟩;
        if w̄_max(i, j) ≥ 0 then
            new_b ← i;
return H;
```

Algorithm A.3: MIG^{++}

```
Data: C = ⟨g_1, ... g_n⟩
Input: win
Result: H = ∅
W ← ⟨0, ..., 0⟩ ;
S ← ⟨0, ..., 0⟩ ;
T ← ⟨2, ..., n⟩ ;
B ← ⟨2, ..., n⟩ ;
new_win ← 0 ;
calculations ← 1;
while calculations > 0 do
    new_win ← new_win + win;
    calculations ← 0;
    b ← 1;
    new_b ← 1;
    for j = 2 to n do
        if new_b = B[j − 1] then
            B[j − 1] ← b;
            b ← T[j − 1];
            new_b ← T[j − 1];
            continue;
        if i − new_b > new_win then
            B[j − 1] ← j − new_win;
            b ← j − new_win;
        else
            B[j − 1] ← b;
            b ← new_b;
        new_b ← T[j − 1];
        for  i = T[j − 1] − 1 downto b do
            w ← w(i, j);
            S[j − 1] ← S[j − 1] + w;
            W[i] ← W[i] + S[j − 1];
            if w = 1 − d and W[i] ≥ 0 then
                H ← H ∪ ⟨g_i, ..., g_j⟩;
            if w̄_max(i, j) ≥ 0 then
                new_b ← i;
            calculations ← calculations + 1;
        T[j − 1] ← b;
return H;
```

(a) HapMapII

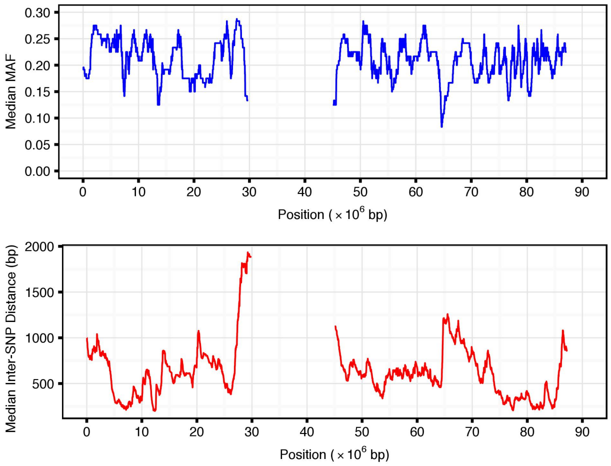

(b) 1000G

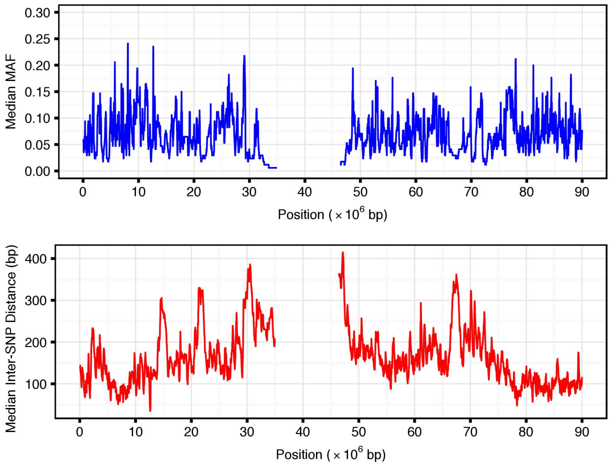

Figure A.1: Median MAF and median inter-SNP distance in sliding regions of 1,000 SNPs on chromosome 16 with no centromere. Similar figures were obtained from all the rest of autosomal chromosomes.

(a) HapMapII

Nr	Genomic Coordinates	Median MAF	Median Inter-SNP Distance (bp)
1	chr8:16110095-16887851	0.0750000	348
2	chr15:40771215-43225829	0.0666667	1207
3	chr16:29576305-34968118	0.1333330	1888
4	chr11:5183617-5559739	0.2291670	163
5	chr20:31767872-33700401	0.2291670	1195
6	chr16:29171648-34806365	0.2000000	1871
7	chr2:15048665-15822444	0.3750000	381
8	chr2:192944318-195085355	0.3916670	1033
9	chr346957741-50137295	0.3458330	2271

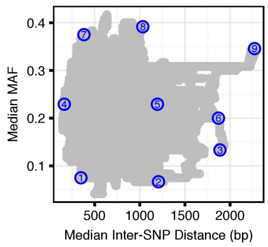

(b) 1000G

Nr	Genomic Coordinates	Median MAF	Median Inter-SNP Distance (bp)
1	chr2:89153688-89307566	0.01176470	42
2	chr16:33617236-34357869	0.00588235	237
3	chr8:48335240-48987674	0.01764710	452
4	chr16:12647838-12700632	0.23529400	35
5	chr13:82569406-82944099	0.24117600	237
6	chr9:66847609-69229598	0.07647060	414
7	chr3:97879829-97981482	0.46470600	57
8	chr13:64621522-64855566	0.43529400	156
9	chr13:64601186-64844532	0.41176500	165

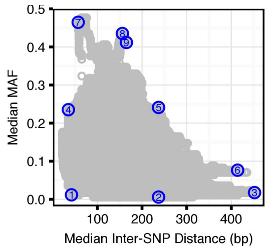

Figure A.2: Sampled regions of 1,000 SNPs. The same sampling method was used for regions of 5,000, 10,000, 15,000, 20,000, 25,000 and 30,000 SNPs. Gray points correspond to all generated regions, while blue points correspond to sampled regions.

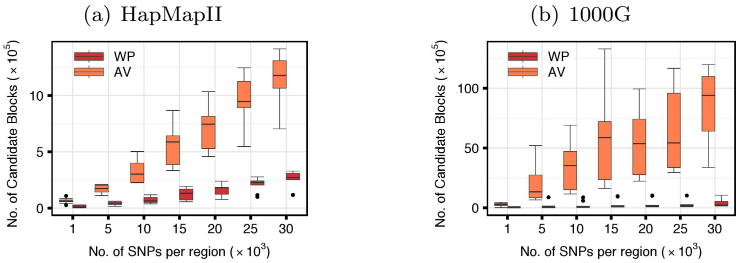

Figure A.3: Number of candidate haplotype blocks detected by the MIG, MIG$^+$ and MIG^{++} algorithms with the WP and AV methods.

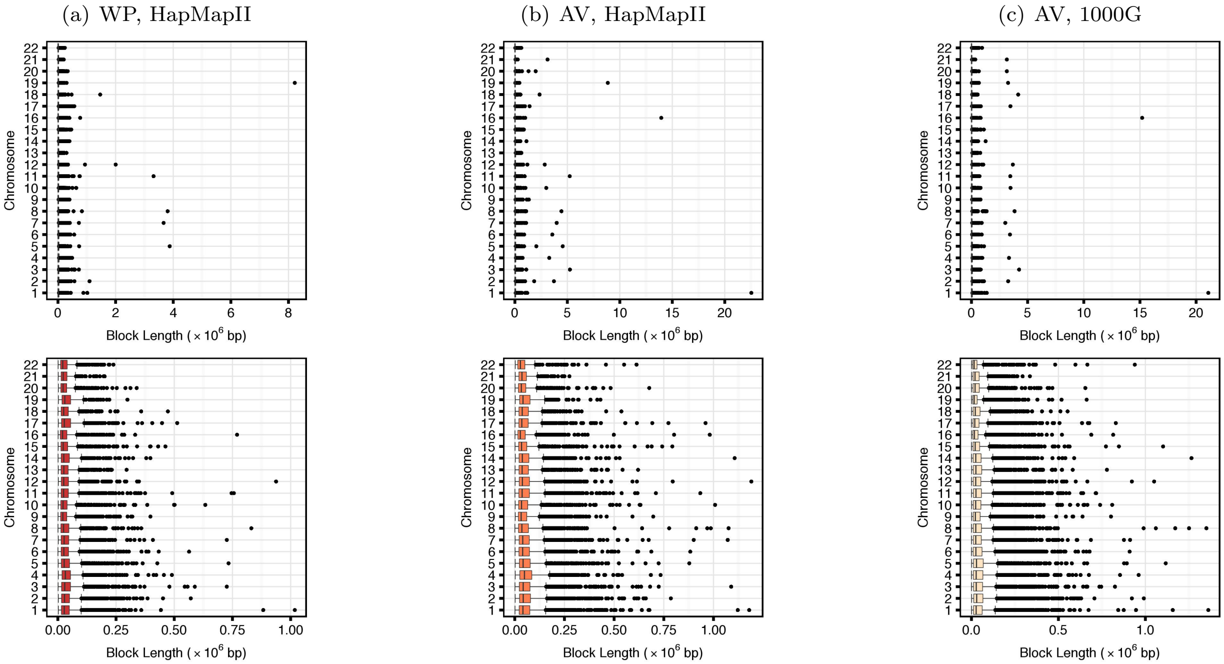

Figure A.4: Lengths of the blocks in base pairs (bp) estimated with the WP and AV methods on the complete HapMapII and 1000G datasets. The figures in the top row plot the length of all haplotype blocks. The figures in the bottom row consider only blocks that do not overlap with centromeres and have a maximal distance between nearby SNPs of at most 1/5 of the total block length.
